# Pediatric external hemorrhoids: clinical characteristics and outcomes of conservative treatment versus injection sclerotherapy

**DOI:** 10.1007/s00431-025-06392-2

**Published:** 2025-08-14

**Authors:** Daniël Docter, Hendrik van Braak, Brenda de Jong, Ramon R. Gorter, Marc A. Benninga, Justin R. de Jong

**Affiliations:** 1 Department of Pediatric Surgery, Emma Children’s Hospital, Amsterdam University Medical Centers, Meibergdreef 9, 1105AZ Amsterdam, The Netherlands; 2 Department of Pediatric Gastroenterology, Emma Children’s Hospital, Amsterdam University Medical Centers, Meibergdreef 9, 1105AZ Amsterdam, The Netherlands

**Keywords:** Pediatric external hemorrhoids, Injection sclerotherapy, Conservative management, Parent-provided photographs

## Abstract

Pediatric external hemorrhoids are rare and often misdiagnosed. This study assesses clinical presentation, risk factors, and treatment outcomes, comparing conservative management with injection sclerotherapy. A retrospective cohort study (2007–2024) was conducted at a tertiary pediatric hospital, including pediatric patients treated with conservative therapy (watchful waiting) or injection sclerotherapy for external hemorrhoids. Data from medical records were analyzed for patient history, presentation, treatment outcomes, and complications. Forty-four patients (86.4% male) were included. Mean age at symptom onset and presentation was respectively 4.0 (range 0–11) and 5.8 (range 2–13) years, with a median diagnostic delay of 20.5 months. All reported anal protrusion/swelling during/after defecation, though this was only visible on examination in 11.4%; diagnosis was otherwise based on parent-provided photographs (63.6%) or reference images (25.0%). Additional symptoms included pain (45.5%), bleeding (18.2%), anal itching (13.6%), anxiety (9.1%) and tenesmus (6.8%). Thirty-one patients (70.5%) underwent injection sclerotherapy, with a 69.2% success rate after one, and 90.3% after two injections. Thirteen patients (29.5%) were treated conservatively with watchful waiting, with symptoms resolving within one year, although the lesion(s) persisted. Skin erosion occurred in 35.5% of patients treated with injection sclerotherapy, leading to constipation and defecation anxiety in 9.1% of patients.

*Conclusion*: Pediatric external hemorrhoids present as anal protrusion/swelling, often accompanied by anorectal pain. Diagnosis is often delayed due to its intermittent nature. Parent-provided photographs can confirm diagnosis. Both conservative management and injection sclerotherapy are viable treatments; injection sclerotherapy offers a definitive resolution but carries risks of relapse and complications.

**Table:** 

**What is Known:** • *Pediatric external hemorrhoids are often misdiagnosed and mismanaged.*
**What is New:** • *Due to the intermittent nature of external hemorrhoids, diagnosis is often delayed, making parent-provided photographs highly valuable for accurate assessment.* • *Both conservative management and injection sclerotherapy are viable treatments.* • *Injection sclerotherapy carries risks of relapse and complications.*

**Supplementary Information:**

The online version contains supplementary material available at 10.1007/s00431-025-06392-2.

## Introduction

Although rare and with an unknown incidence, external hemorrhoids (EH) represent a distinct anorectal condition in the pediatric population. Consequently, EH are often misdiagnosed and mismanaged [1–3].

**Fig. 1:**
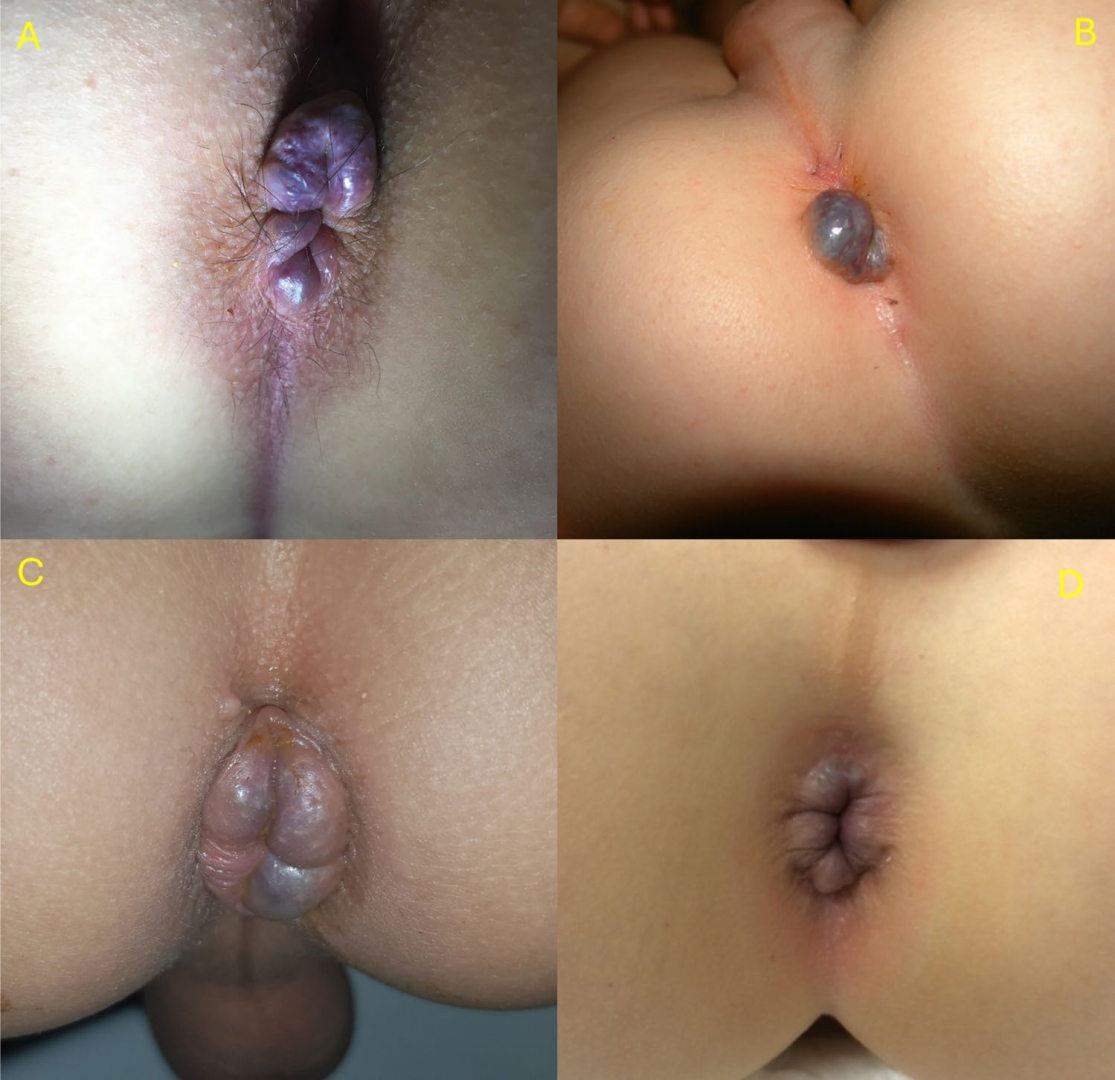
Four cases of external hemorrhoids, showing the typical bulging veins of the perianal vessels. They can present as bulging (**A**, **B**) or thickened (**C**, **D**)

EH arise below the dentate line as bulging veins of the perianal vessels on the anal verge, are innervated by cutaneous nerves and covered in skin [2, 4–7]. This in contrast to internal hemorrhoids (IH), which arise above the dentate line from the internal hemorrhoidal plexus, are innervated by visceral nerves and are covered by mucosa [5, 8, 9]. Furthermore, IH rarely protrude and do not occur in children. Presentation of EH in children is similar to adults; a protruding blue lesion around the anus which can be painful in case of thrombosis (Fig. 1) [1, 8, 10].

Management is primarily conservative, focused on dietary and lifestyle modifications. The EH typically resolve spontaneously within one to two weeks, occasionally leaving behind a skin tag [1–3]. EH can also be treated using injection sclerotherapy (IS) [2], while surgical excision, previously considered in rare cases of thrombosed EH, is no longer part of standard practice [1, 11]. Still, these management approaches are not specifically tailored to children; they are largely extrapolated from adult studies, as literature on pediatric EH remains scarce [11, 12].

This study aims to assess the clinical presentation and risk factors of EH, and to evaluate treatment outcomes by comparing conservative management with IS.

## Materials and methods

### Patients and study design

We conducted a single-center retrospective cohort study of patients (< 18 years) who were treated for EH at the Emma Children’s Hospital, Amsterdam University Medical Centers, Department of Pediatric Surgery between 2007 and 2024. EH was diagnosed either through physical examination or by reviewing digital photographs or videos sent by parents or pediatricians. No additional work-up was required for this diagnosis.

### Data extraction

Data were retrospectively obtained from patient records. Symptom resolution was defined as the absence of any EH-related discomfort or interference in daily life. EH was considered resolved when no swelling was visible following treatment, aside from any remaining skin tags.

### Treatment protocol

Parents were informed by the pediatric surgeon about the benign nature of EH and its potential for spontaneous resolution. Both conservative management and IS were presented as valid options, with IS offering a higher chance of definitive resolution. Patients were informed about the possible complications of IS treatment, including standard surgical complications as well as skin erosions specifically associated with IS. Final treatment decisions were made by parents after considering these factors.

### Conservative treatment

For patients managed with conservative treatment (watchful waiting, lifestyle and dietary advises), two follow-up options were offered: either scheduled annual consultations by phone to monitor the EH, or no routine follow-up, with the agreement that parents would contact the outpatient clinic if symptoms persisted or worsened. Laxatives are not included in our standard therapeutic approach for EH.

### Injection sclerotherapy (IS)

**Fig. 2:**
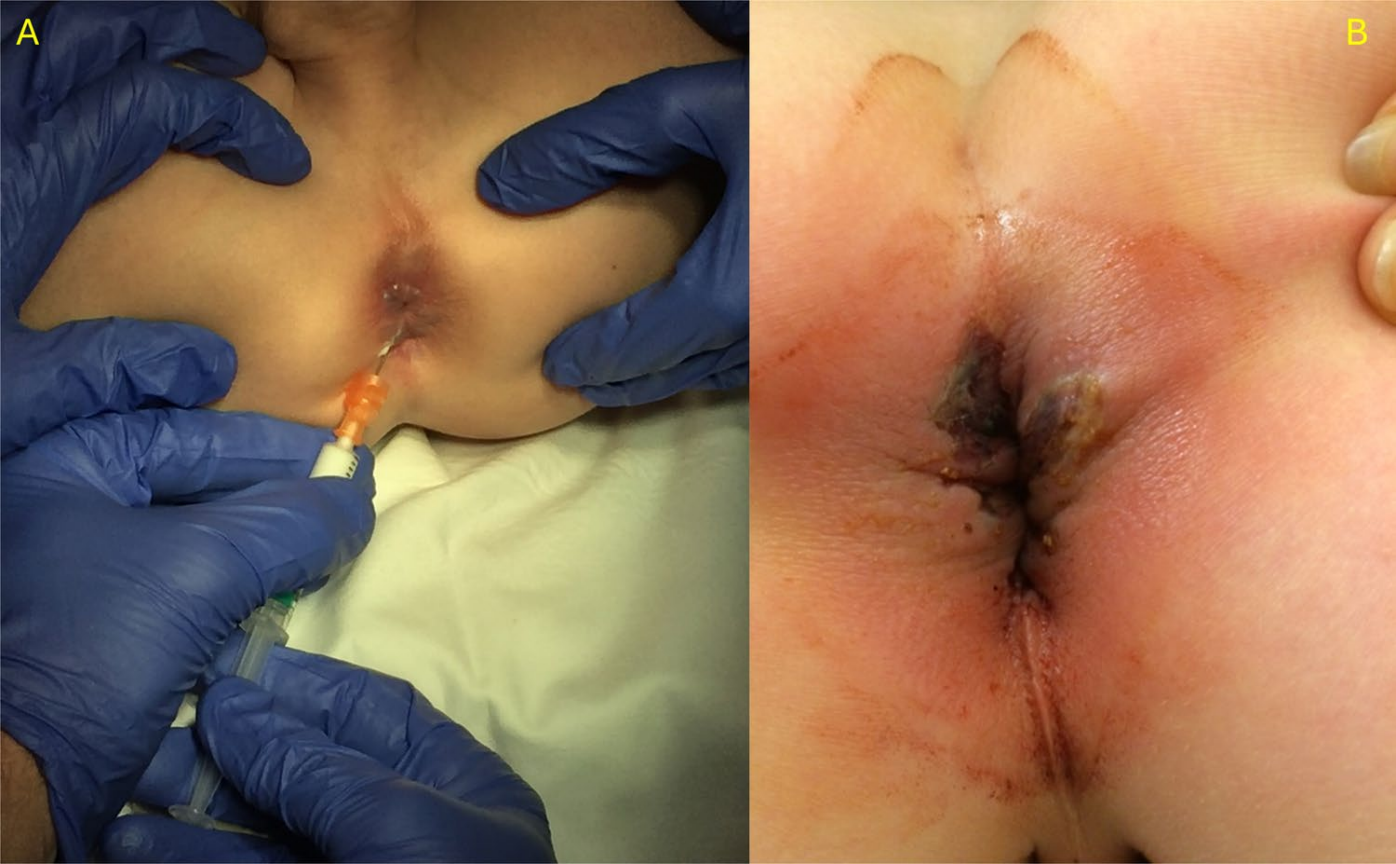
Injection of sclerotherapy (2% Ethoxysclerol) in the veins of the external hemorrhoid (**A**). The most common complication of this procedure is skin erosion (**B**)

IS (Fig. 2) was performed under full anesthesia, with the patient in supine position and legs up in braces. EH was confirmed by applying abdominal pressure to distend the hemorrhoidal plexus. Dilated veins were injected with Ethoxysclerol solution 2% (Lauromacrogol 400, 20 mg/ml) to destroy vasculature. Patients were discharged within 24 h. Treatment was repeated in case of recurrence until symptoms resolved or patients did not want to proceed with treatment. Skin erosion, a common complication, was treated with silver-sulfadiazine sterile ointment 1% (Flammazine®).

### Outcomes

The primary outcome was resolution of patients symptoms. Secondary outcomes included the assessment of clinical presentation and history of patients (age at symptom onset, age at presentation, reported symptoms, history of diarrhea or constipation) and complications of IS.

### Statistical analysis

Descriptive measurements were utilized to characterize the study population. Continuous variables were summarized as mean ± standard deviation (SD) if normally distributed, or as median with interquartile range (IQR) if not. Dichotomous variables were summarized using frequencies and percentages. Statistical significance was set at P < 0.05. Data were analyzed using IBM SPSS Statistics 28.0.

## Results

**Fig. 3:**
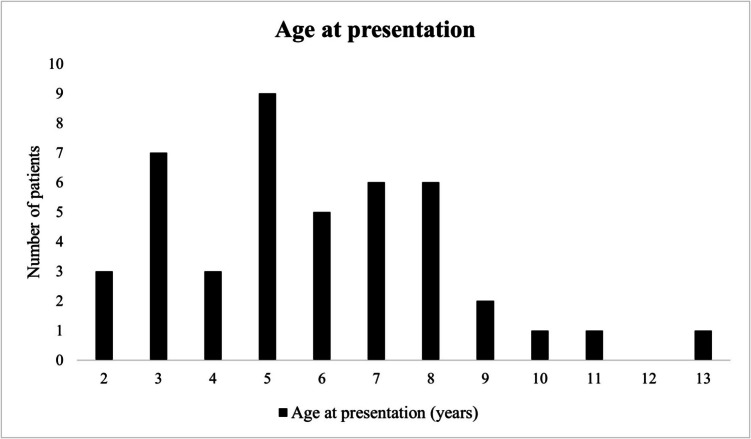
Distribution of patients by age of clinical presentation

A total of 44 patients were treated for EH during an inclusion period of seventeen years. Of these patients, 86.4% (n = 38/44) were male. The mean age at presentation was 5.8 (range 2–13) years, or 75.6 (range 30–164) months (Fig. 3). Median follow-up was 62.0 (IQR 46.5–124.5) months. Table 1 depicts baseline characteristics and patient history. A significant proportion of patients (13.6%, n = 6/44) had been previously misdiagnosed as IH, and 6.8% (n = 3/44) had undergone prior treatment with rubber band ligation at another center. At our clinic, the majority of patients 70.5% (n = 31/44) were treated with IS, and 29.5% (n = 13/44) received conservative treatment.

**Table 1:** Baseline characteristics and symptoms

	Overall (N = 44)	Conservative (n = 13/44)	IS (n = 31/44)	p-value
Age at presentation (m)	75.6 (30.4)	71.3 (28.3)	77.4 (31.5)	0.54
Age at symptoms onset (m)	48.1 (31.0)	48.4 (19.3)	47.9 (35.1)	0.96
Time between symptom onset and presentation (m)^a^	20.5 (8.3–36.0)	12.0 (5.0–37.5)	23.0 (9.0–36.0)	0.47
Follow-up (m)^a^	62.0 (46.5–124.5)	116.0 (47.0–159.0)	58.0 (46.0–104.0)	0.26
Sex^b^				>.99
* Male*	38 (86.4)	11 (84.6)	27 (87.1)	
* Female*	6 (13.6)	2 (15.4)	4 (12.9)	
Positive family history ^b^	2 (4.5)	1 (7.7)	1 (3.2)	0.51
Patient history ^b^				
*Constipation*	9 (20.5)	1 (7.7)	8 (25.8)	0.24
* Diarrhea*	15 (34.1)	3 (23.1)	12 (38.7)	0.49
*Use of stool softeners*	9 (20.5)	2 (15.4)	7 (22.6)	0.70
*Lactose intolerance*	3 (6.8)	0 (0.0)	3 (9.7)	0.54
* Previous misdiagnosis*	6 (13.6)	2 (15.4)	4 12.9)	>.99
*Previous rubber band ligation*	3 (6.8)	0 (0.0)	3 (9.7)	0.54
* Sexual abuse*	1 (2.3)	0 (0.0)	1 (3.2)	0.51
Symptoms^b^				
*Anal protrusion/swelling*	44 (100.0)	13 (100.0)	31 (100.0)	n/a
*Progressive*	7 (15.9)	1 (7.7)	6 (19.4)	0.65
*Pain*	20 (45.5)	3 (23.1)	17 (54.8)	0.05
*Bleeding*	8 (18.2)	0 (0.0)	8 (25.8)	0.08
*Anal itching*	6 (13.6)	3 (23.1)	3 (9.7)	0.34
*Anxiety related to the visible protrusion/swelling*	4 (9.1)	1 (7.7)	3 (9.7)	>.99
*Tenesmus*	3 (6.8)	1 (7.7)	2 (6.5)	>.99
*Thrombosed EH*	1 (2.3)	0 (0.0)	1 (3.2)	0.51
*Parent reported hygiene difficulties *^b^	11 (25.0)	2 (15.4)	9 (29.0)	0.46

^a^Continues variables expressed as median (IQR); all remaining continuous variables are expressed as mean ± standard deviation

^b^Data displayed as n (%)

IQR = Interquartile Range, IS = injection sclerotherapy, m = months, n/N = number

Patients reported reasons for choosing IS over conservative treatment included difficulty maintaining hygiene after defecation due to the swelling or painful wiping, and parental or child anxiety triggered by the presence of blood on toilet paper. There were no significant differences between the conservatively managed group and the IS-treated group with respect to the baseline characteristics or patient history.

### Age at symptom onset and at presentation, reported symptoms

**Fig. 4:**
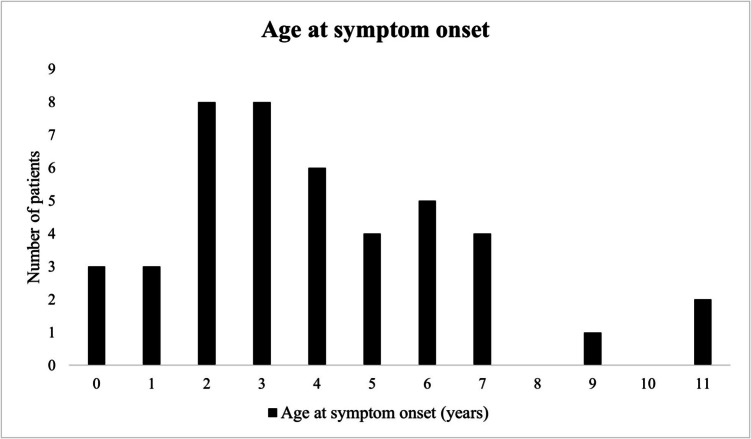
Distribution of patients by age at symptom onset

Median time between symptom onset and presentation was 20.5 months (IQR 8.3–36), with the swelling typically first appearing at a mean age of 4.0 (SD 2.8) years, or 48.1 (SD 31.0) months. In 13.6% (n = 6/44) of patients the EH had been present since birth, or arose during the first year of life (Fig. 4).

All patients reported anal protrusion/swelling visible during or directly after defecation, however, in only 11.4% of patients (n = 5/44), the swelling was visible during physical examination. In 63.6% of cases (n = 28/44), the diagnosis was based on photographs provided by the parents, while in 25.0% (n = 11/44), the parents identified the external hemorrhoid after being shown reference images by the surgeon. In 20.5% of patients (n = 9/44), swelling was the only reported symptom and in 15.9% of patients (n = 7/44) the protrusion/swelling was noted to be progressive over time. Additional symptoms included pain (45.5%, n = 20/44), bleeding (18.2%, n = 8/44), anal itching (13.6%, n = 6/44), anxiety related to the visible protrusion/swelling (9.1%, n = 4/44) and tenesmus (6.8%, n = 3/44). In 25.5% of patients (n = 11/44), parents reported difficulty in maintaining their child’s hygiene after defecation due to the presence of the swelling.

Thrombosis of EH was identified in only one case (2.3%), based on parental photographs showing a previously thrombosed lesion. At the time of presentation, however, no thrombosis was observed. In all other cases, there was no evidence of thrombosed EH at presentation or in the patient's history.

There were no significant differences between the conservatively managed group and the IS-treated group with respect to age at symptom onset, presentation and reported symptoms (Table 1).

### Treatment outcomes

Following the initial consultation and counseling, 46.2% (n = 6/13) of conservatively managed patients did not return to the outpatient clinic. The remaining 53.8% (n = 7/13) were contacted by phone after one year; all reported symptom resolution within that year, and no further interventions were required. Of the patients managed with IS, 30.8% (n = 8/31) experienced relapse of EH. Six of these patients needed a second injection of IS because of persisting symptoms, which was successful in five cases. The remaining patient required three additional injections before the symptoms resolved. The other two patients with relapse opted not to pursue further treatment.

Overall, the success rate after one round of IS was 69.2% (n = 23/31), increasing to 90.3% (n = 28/31) after two rounds and 93.5% (n = 29/31) after five rounds.

### Complications of IS

At the two-month follow-up, 35.5% (n = 11/31) reported skin erosion due to IS. Most of these erosions were successfully managed with Flammazine®, with the skin erosion resolving within six months, except for one patient. This patient developed anal fissures as a complication of the skin erosion, which required surgical intervention. The fissures were sutured, and 25 units of botulinum toxin (Botox) were injected into the internal anal sphincter. This successfully resolved the symptoms. Although skin erosion was effectively treated in all cases, it was often painful and led, in four (9.1%) patients, to persistent troublesome defecation even after healing. Two patients temporarily required stool softeners for constipation, one patient developed defecation anxiety, requiring referral to the pediatric gastroenterologist. Another patient showed abnormal toileting behavior and began avoiding sitting and started urinating and defecating while standing, necessitating pelvic floor therapy.

## Discussion

This is the first study to investigate the use of IS for EH in pediatric patients. Our results demonstrate that IS is an effective treatment option for pediatric EH, with a resolution rate of 93.5%. Relapse occurred in nearly one-third of IS-treated patients, but most patients responded well to a second round of treatment. Skin erosion was the only complication of IS (35.5%), but resulted in a significant part of patients developing troublesome defecation. Conservative management and adequate counseling led to resolution of symptoms in all cases, although the EH itself remained present.

### Delay of diagnosis, age at symptom onset and at presentation

We found a median delay of 20.5 months from symptom onset to diagnosis of EH. Additionally, a notable proportion of patients had been previously misdiagnosed or received inappropriate treatment. These findings highlight the need for greater awareness of pediatric EH among healthcare providers to improve timely and accurate diagnosis. Since EH was usually not visible during physical examination, photos provided by parents proved to be a highly reliable tool for confirming the diagnosis [13].

In contrast with our findings, the scarcely available studies on EH reported an later age of presentation (11.7 years and six, ten and twelve years) compared to our patients (four years) [11, 14]. The reason for this discrepancy is unclear, but it may be related to higher parental awareness in our population or delayed diagnosis in their population. If the true age of onset is indeed earlier than previously assumed, this challenges the widely held belief that pediatric EH primarily develop during adolescence [2, 3, 14]. In our cohort, the peak incidence occurred before the age of four, aligning with toilet training milestones, suggesting that defecation habits, such as straining and prolonged sitting, may be contributing factors. Interestingly, one study found a significant association between bidet use and EH, indicating a possible environmental factor [11]. In addition, a notable proportion of our patients had EH present from birth or developed EH within the first year of life. This finding raises the possibility of a congenital component in pediatric EH, particularly given the typically dense and resilient nature of anorectal connective tissue in early childhood.

Although literature describes a possible association between EH, chronic liver failure and portal vein hypertension [3, 15], the incidence of both EH and these underlying conditions is very low. In this study, no patients had a history of portal vein hypertension or liver disease. Given the rarity of both conditions, routine screening for liver disease in children presenting with EH does not appear to be warranted.

### Influence of patient history and sex on EH

Constipation was present in 21% of our patients, nearly twice the prevalence reported in the general pediatric population [16], supporting its role as a relevant risk factor [1, 2, 11]. Interestingly, a larger proportion of patients (34%) had a history of diarrhea, aligning with adult studies that suggest diarrhea may also play a role in the development of hemorrhoids. This is thought to result from prolonged contraction of the anal sphincter to maintain fecal continence, leading to elevated anal resting pressure, a factor associated with hemorrhoid development [17]. This finding challenges the traditional focus on constipation only and suggest that the routine prescription of stool softeners may not be appropriate in all cases. Pediatric EH requires tailored conservative treatment based on individual bowel patterns without assumption that constipation is the only primary cause.

In accordance with the literature, the majority of patients in our study were male [11, 14]. A possible explanation for this sex difference is the anatomical difference in anal canal length, which tends to be longer in males [18]. This may influence venous drainage and lead to increased venous stasis, thereby raising the risk of developing EH.

### Symptoms and risk factors

While thrombosed EH is frequently seen in adults, it appears to be less common in pediatric patients, although one study reported a 23% incidence in children. We observed only one case of possible thrombosed EH in our series [11]. The discrepancy with the adult population may be due to difference in risk factors, such as smoking, alcohol, or pregnancy, which are absent in children. The absence of these factors could partly explain why thrombosis appears to occur less frequently in the pediatric population. Additionally, increased parental vigilance may lead to earlier recognition of non-thrombosed EH in children, whereas in adults, such cases might go unnoticed without thrombosis, resulting in underreporting of non-thrombosed EH in adult studies. We have no clear explanation for the discrepancy in thrombosis rates between the referenced study on pediatric EH and our cohort [11]. In our experience, thrombosed EH are rarely observed in pediatric patients.

Anoreceptive sex is also a risk factor for EH [19]. The presence of one confirmed case of sexual abuse in our cohort highlights the need for careful assessment when children present with anorectal complaints, including benign conditions like EH.

The incidence of other symptoms, including swelling, pain, bleeding, and itching, was consistent with previously reported findings in the literature [11].

### Outcomes of treatment

To date, data are lacking on the use of IS for the treatment of external EH in children. Even in adults the effect of IS is not well studied, with a wide range (20%−88%) of published success [20]. We found a comparable resolution rate of 90.3% after two rounds of IS, and 93.5% after five rounds of IS. However, relapse remains a challenge, with 30.8% of patients experiencing recurrence of EH. Most patients underwent a second round of IS, requiring an additional procedure under general anesthesia. This illustrates that while IS is minimally invasive, its high relapse rate may necessitate repeated treatments, resulting in multiple exposures to general anesthesia.

In line with the current literature in adults with EH, conservative management led to spontaneous resolution of symptoms in all cases, with percentages varying from 25.4% to 100% [11, 21, 22].

### Complications

One-third of patients developed post-operative skin erosion, which had a significant impact on daily functioning. In young children, especially, painful complications may result in altered toileting habits or withholding behavior, which can be difficult to manage and may require multidisciplinary follow-up. This underlines the importance of adequately counseling parents about possible complications, not only to manage expectations but also to ensure timely recognition and support if defecation-related issues arise during recovery.

### Limitations

This study was limited by its retrospective design and small cohort size. As a result, we were unable to evaluate several potential risk factors for pediatric EH, such as obesity, low fiber intake, skipping breakfast, type of toilet (access to bidet), and prolonged toilet sitting times, since these were not routinely discussed or recorded during consultations.

Additionally, the small sample size limited the statistical power of our analyses, particularly when comparing outcomes between conservative treatment and IS.

### Conclusions

Pediatric patients with EH typically present with anal protrusion or swelling, often accompanied by pain. When EH is not visible during physical examination, parent-provided photographs are of major importance to confirm the diagnosis. The early age of onset observed in our cohort challenges the prevailing assumption that EH primarily develops during adolescence and suggests a potential role for congenital factors. The frequent diagnostic delays, likely due to the intermittent nature of EH, highlight the need for greater awareness among healthcare providers.

Conservative management, based on a watchful waiting approach and supported by adequate counseling, was effective in relieving parental distress, with symptoms resolving within a year; however, EH itself did not resolve completely. IS proved to be a safe and effective treatment with a high resolution rate; however, recurrence and complications remain a challenge.

## Supplementary Information

Below is the link to the electronic supplementary material.

Supplementary file1 (DOCX 33.2 KB)

## Data Availability

No datasets were generated or analysed during the current study.

## Abbreviations

EH: External hemorrhoids
IH: Internal hemorrhoids
IS: Injection sclerotherapy
